# A Public Health Framework for Legalized Retail Marijuana Based on the US Experience: Avoiding a New Tobacco Industry

**DOI:** 10.1371/journal.pmed.1002131

**Published:** 2016-09-27

**Authors:** Rachel Ann Barry, Stanton Glantz

**Affiliations:** 1 Center for Tobacco Control Research and Education and Philip R. Lee Institute for Health Policy Studies, University of California, San Francisco, San Francisco, California, United States of America; 2 Department of Medicine, University of California, San Francisco, San Francisco, California, United States of America

## Abstract

Rachel Barry and Stanton Glantz argue that a public health framework that prioritizes public health over business interests should be used by US states and countries that legalize retail marijuana.

**Summary Points**

- The US states that have legalized retail marijuana are using US alcohol policies as a model for regulating retail marijuana, which prioritizes business interests over public health.
- The history of major multinational corporations using aggressive marketing strategies to increase and sustain tobacco and alcohol use illustrates the risks of corporate domination of a legalized marijuana market.
- To protect public health, marijuana should be treated like tobacco, not as the US treats alcohol: legal but subject to a robust demand reduction program modeled on successful evidence-based tobacco control programs.
- Because marijuana is illegal in most places, jurisdictions worldwide (including other US states) considering legalization can learn from the US experience to shape regulations that prioritize public health over profits.

## Introduction

While illegal in the United States, marijuana use has been increasing since 2007 [1]. In response to political campaigns to legalize retail sales, by 2016 four US states (Colorado, Washington, Alaska, and Oregon) had enacted citizen initiatives to implement regulatory frameworks for marijuana, modeled on US alcohol policies [2], where state agencies issue licenses to and regulate private marijuana businesses [2,3,4]. Arguments for legalization have stressed the negative impact marijuana criminalization has had on social justice, public safety, and the economy [5]. Uruguay, an international leader in tobacco control [6], became the first country to legalize the sale of marijuana in 2014, and, as of July 2016, was implementing a state monopoly for marijuana production and distribution [7]. None of the US laws [2], or pending proposals in other states [8], prioritize public health. Because marijuana is illegal in most places, jurisdictions worldwide (including other US states) considering legalization can learn from the US experience to shape regulations that favor public health over profits.

In contrast, while legal, US tobacco use has been declining [1]. To protect public health, marijuana should be treated like tobacco, legal but subject to a robust demand reduction program modeled on evidence-based tobacco control programs [9] before a large industry (akin to tobacco [10]) develops and takes control of the market and regulatory environment [11].

### Likely Effect of Marijuana Commercialization on Public Health

While the harms of marijuana do not currently approach those of tobacco [12], the extent to which legal restrictions on marijuana may have functioned to limit these harms is unknown. Currently, regular heavy marijuana use is uncommon, and few users become lifetime marijuana smokers [13]. However, marijuana use is not without risk. The risk for developing marijuana dependence (25%) is lower than for nicotine addiction (67%) and higher than for alcohol dependence (16%) [14], but is still substantial, with rising numbers of marijuana users in high-income countries seeking treatment [15]. Reversing the historic pattern, in some places, marijuana has become a gateway to tobacco and nicotine addiction [15].

This situation will likely change as legal barriers that have kept major corporations out of the market [10] are removed. Unlike small-scale growers and marijuana retailers, large corporations seek profits through consolidation, market expansion, product engineering, international branding, and promotion of heavy use to maximize sales, and use lobbying, campaign contributions, and public relations to create a favorable regulatory environment [2,11,16,17,18,19]. By 2016, US marijuana companies had developed highly potent products [15] and were advertising via the Internet [11] and developing marketing strategies to rebrand marijuana for a more sophisticated audience [20]. Without effective controls in place, it is likely that a large marijuana industry, akin to tobacco and alcohol, will quickly emerge and work to manipulate regulatory frameworks and use aggressive marketing strategies to increase and sustain marijuana use [10,11] with a corresponding increase in social and health costs.

Public perception of the low risk of marijuana [21] is discordant with available evidence. Marijuana smoke has a similar toxicity profile as tobacco smoke [22] and, regardless of whether marijuana is more or less dangerous than tobacco, it is not harmless [2]. The California Environmental Protection Agency has identified marijuana smoke as a cause of cancer [23], and marijuana smokers are at increased risk of respiratory disease [24,25]. Epidemiological studies in Europe have found associations between smoking marijuana and increased risk of cardiovascular disease, heart attack, and stroke in young adults [15,26]. One minute of exposure to marijuana smoke significantly impairs vascular function in a rat model [27]. In humans, impaired vascular function is associated with adverse cardiovascular outcomes including atherosclerosis and myocardial infarction [27,28,29].

Acute risks associated with highly potent marijuana products (i.e., cannabinoid concentrates, edibles) include anxiety, panic attacks, and hallucinations [15]. Other health risks associated with use include long-lasting detrimental changes in cognitive function [13,15], poor educational outcomes, accidental childhood ingestion and adult intoxication [26], and auto fatalities [30,31].

### US Alcohol Policy Is Not a Good Model for Regulating Marijuana

The fact that US marijuana legalization is modeled on US alcohol policies is not reassuring. In 2014, 61% of US college students (age 18–25) reported using alcohol in the past 30 days, compared to 19% for marijuana and 13% for tobacco [32]. Binge drinking is a serious problem, with 41% of young Americans reporting heavy episodic drinking in the past year [33].

Aggressive alcohol marketing likely contributes to this pattern [34]. Even though the alcohol industry’s voluntary rules prohibit advertising on broadcast, cable, radio, print, and digital communications if more than 30% of the audience is under age 21, this standard permits them to advertise in media outlets with substantial youth audiences [35], including *Sports Illustrated* and *Rolling Stone*, resulting in American youth (ages 12–20) being exposed to 45% more beer and 27% more spirits advertisements than legal drinking-aged adults [36]. If such alcohol marketing regulations were applied universally to marijuana, consumption would likely be higher, not lower, than it is now [26].

### Using a Public Health Framework from Evidence-Based Tobacco Control to Regulate Retail Marijuana

Table 1 compares the situation in the four US states that have legalized retail marijuana to a public health standard based on successes and failures in tobacco and alcohol control. A public health framework for marijuana legalization would designate the health department as the lead agency with, like tobacco, a mandate to protect the public by minimizing all (not just youth) use. The health department would implement policies to protect nonusers, prevent initiation, and encourage users to quit, as well as regulate the manufacturing, marketing, and distribution of marijuana products, with other agencies (such as tax authorities) playing supporting roles.

**Table 1:** Public health framework versus state marijuana regulations.

	*Public Health Standard*	*Colorado*	*Washington*	*Alaska*	*Oregon*
**Lead Agency**					
Department of Health	✓	X	X	X	X
**Advisory Committees**					
Membership solely of public health experts	✓	✓	**--**	X	✓
No decision-making authority for marijuana industry or vested interests	✓	X	**--**	X	X
**Regulatory Complexity**					
Creates a single system of retail marijuana	✓	X	✓	X	X
**Tax Revenue**					
Tax covers full costs	✓	X	X	--	X
Dedicated revenue to marijuana prevention, control, and research	✓	X	✓	--	X
**Prevention and Control Programs**					
***Media campaign***					
Aimed at general population (not just youth)	✓	X	X	--	X
Modeled on social norm change	✓	X	X	--	X
**Smokefree Laws**					
Prohibit any public use of marijuana	✓	✓	✓	✓	✓
Prohibit marijuana use wherever tobacco smoking is prohibited	✓	✓	✓	X	✓
Protect local control	✓	✓	✓	✓	✓
Prohibit indoor use in marijuana retail stores or marijuana clubs	✓	X	✓	X	✓
**Marketing and Advertising**					
Prohibit free or discounted samples	✓	X	✓	✓	✓
Prohibit cartoon characters	✓	✓	✓	✓	✓
Prohibit sport and cultural event sponsorship	✓	X	X	X	X
Prohibit product placement in popular media and cobranded merchandise	✓	X	X	X	X
Prohibit therapeutic claims	✓	✓	✓	✓	✓
Prohibit outdoor advertising on billboards	✓	✓	X	X	X
Prohibit advertising on television and radio	✓	X	✓	X	X
Restrict advertising in print and digital communications with 15% threshold	✓	X	X	X	X
**Licensing Rules**					
Impose serious penalties on retailers’ underage sales	✓	✓	✓	--	✓
Prohibit sale of tobacco or alcohol in marijuana retail stores	✓	✓	✓	✓	✓
Prohibit tobacco and alcohol retailers from holding marijuana license	✓	X	X	X	X
**Retail Sales**					
Require retailer use age verification system (ID scanners) at point of sale	✓	X	X	X	X
Prohibit retailers within 1,000 feet of underage-sensitive areas	✓	X	✓	X	X
Prohibit electronic commerce (internet, mail order, text messaging, social media)	✓	✓	✓	✓	✓
**Product Standards**					
Require strong potency limits and product quality testing	✓	X	X	X	✓
Prohibit products containing additives (nicotine, alcohol, caffeine, or toxic chemicals)	✓	✓	X	✓	✓
Prohibits flavored products appealing to underage persons	✓	X	✓	X	X
**Warning Labels**					
Require warning labels modeled on state of the art tobacco labels	✓	X	X	X	X

**Key:** ✓ Required by law or regulation; X Not required by law or regulation; --Pending legislative approval or rulemaking process

Because public health regulations are often in direct conflict with the interests of profit-driven corporations [19], it is important to protect the policy process from industry influence. In contrast to what states that have legalized retail marijuana have done to date, a public health framework would require that expert advisory committees involved in regulatory oversight and public education policymaking processes consist solely of public health officials and experts and limit the marijuana industry’s role in decision-making to participation as a member of the “public.” Including the tobacco industry on advisory committees when developing tobacco regulations blocks, delays, and weakens public health policies [37]. The World Health Organization Framework Convention on Tobacco Control, a global public health treaty ratified by 180 parties as of April 2016, recognizes the need to protect the policymaking process from industry interference:

“[Governments] should not allow any person employed by the tobacco industry or any entity working to further its interests to be a member of any government body, committee or advisory group that sets or implements tobacco control or public health policy.” [37, Article 5.3]

A marijuana regulatory framework that prioritizes public health would have similar provisions.

A public health framework would avoid regulatory complexity that favors corporations with financial resources to hire lawyers and lobbyists to create and manipulate weak or unenforceable policies [11]. To simplify regulatory efforts, including licensing enforcement, implementation of underage access laws, prevention and education programs, and taxation, a public health framework would create a unitary market, in which all legal sales, regardless of whether use is intended for recreational or medical purposes, follow the same rules [38]. Unlike Colorado, Oregon, and Alaska, in 2015, Washington State accomplished this public health goal when it merged its retail and medical markets [39].

Earmarked funds to support comprehensive prevention and control programs over time, which are not included in the four US states’ regulatory regimes, will be critical to reduce marijuana prevalence, marijuana-related diseases, and costs arising from marijuana use. A public health framework would set taxes high enough to discourage use and cover the full cost of legalization, including a broad-based marijuana prevention and control program. Using a public health approach, the prevention program would implement social norm change strategies, modeled on evidence-based tobacco control programs, aimed at the population as a whole—not just users or youth [9]. Demand reduction strategies applied to marijuana would include: 1) countering pro-marijuana business influence in the community; 2) reducing exposure to secondhand marijuana smoke and aerosol and other marijuana products (including protecting workers vulnerable to these exposures); 3) controlling availability of marijuana and marijuana products; and 4) promoting services to help marijuana users quit.

A public health framework would protect the public from secondhand smoke exposure by including marijuana in existing national and local smokefree laws for tobacco products, including e-cigarettes. Local governments would have authority to adopt stronger regulations than the state or nation. There would be no exemptions for indoor use in hospitality venues, marijuana retail stores, or lounges, including for “vaped” marijuana.

To protect the public from industry strategies to increase and sustain marijuana use, a public health framework would prohibit or severely restrict (within constitutional limitations) marketing and advertising, including prohibitions on free or discounted samples, the use of cartoon characters, event sponsorship, product placement in popular media, cobranded merchandise, and therapeutic claims (unless approved by the government agency that regulates such claims). Marketing would be prohibited on television, radio, billboards, and public transit and restricted in print and digital communications (e.g., internet and social media) with the percentage of youth between ages 12 and 20 as the maximum underage audience composition for permitted advertising (roughly 15% in the US) [35]. These advertising restrictions are justified and would likely pass US Constitutional muster because they are implemented for important public health purposes, are evidence-based [35], and have worked to promote similar goals in other contexts. Legal sellers of the newly legal marijuana products would be permitted to communicate relevant product information to their legal adult customers.

A scenario in which a public health regulatory framework is applied to marijuana would require licensees to pay for strong licensing provisions for retailers, with active enforcement and license revocation for underage sales. As has been done in the four US states (Table 1), outlets would be limited to the sale of marijuana only to avoid the proliferation and normalization of sales in convenience stores or “big box” retailers. No retailer that sold tobacco or alcohol would be granted a license to sell marijuana products. Based on best public health practices for tobacco retailers [40], marijuana retail stores would be prohibited within 1,000 feet of underage-sensitive areas including postsecondary schools, with limits on new licenses in areas that already have a significant number of retail outlets. Electronic commerce, including internet, mail order, text messaging, and social media sales, would be prohibited because these forms of nontraditional sales are difficult to regulate, age-verification is practically impossible [41], and they can easily avoid taxation [42].

Central to a public health framework would be assigning the health department with the authority to enact strong potency limits, dosage, serving size, and product quality testing for marijuana and marijuana products (e.g., edibles, tinctures, oils), with a clear mission to protect public health. Additives that could increase potency, toxicity, or addictive potential, or that would create unsafe combinations with other psychoactive substances, including nicotine and alcohol, would be illegal. Unlike US restrictions on marijuana products, flavors (that largely appeal to children), would be prohibited.

A public health model applied to marijuana would include health warning labels that follow state-of-the-art tobacco requirements implemented in several countries outside of the United States, including Uruguay, Brazil, Canada, and Australia [43]. Public health-oriented labels would: 1) be large, (at least 50% of packaging) on front and back and not limited to the sides, prominently featured, and contain dissuasive imagery in addition to text; 2) be clear and direct and communicate accurate information to the user regarding health risks associated with marijuana use and secondhand exposure; and 3) use language appropriate for low-literacy adults. Health messages would include risk of dependence [2], cardiovascular [2,44,45], respiratory [25], and neurological disease [46], and cancer [23], and would warn against driving a vehicle or operating equipment, as well as the risks of co-use with tobacco or alcohol.

While there is already adequate scientific evidence to raise concern about a wide range of adverse health effects, there is more to learn. Earmarked funds from marijuana taxes would also provide an ongoing revenue stream for research that would guide marijuana prevention and control efforts and mitigate the human and economic costs of marijuana use, as well as better define medical uses as the basis for proper regulation of marijuana for therapeutic purposes.

### Avoiding a Private Market

Privatizing tobacco and alcohol sales leads to intensified marketing efforts, lower prices, more effective distribution, and an industry that will aggressively oppose any public health effort to control use [47,48]. Avoiding a privatized marijuana market and the associated pressures to increase consumption in order to maximize profits would likely lead to lower consumer demand, consumption, and prevalence, even among youth, and would reduce the associated public health harm [49].

Governments may avoid marijuana commercialization by implementing a state monopoly over its production and distribution, similar to Uruguay’s regulatory structure for marijuana [3,50] and to the Nordic countries’ alcohol control systems [51], which are designed to protect public health over maximizing government revenue. The state would have more control over access, price, and product characteristics (including youth-appealing products or packaging, potency, and additives) and would refrain from marketing that promotes increased use [3,52]. In cases where national laws cause concern about local authority’s ability to adopt government monopolies, a public health authority could be used as an alternative [53].

It is important to avoid intrinsic conflicts of interest created by state ownership. As is the case with state-ownership of tobacco, without specific policies to prioritize public health, a state’s desire to increase revenue often supersedes public health goals to minimize use [51,52]. Beyond mitigating potential conflicts of interest inherent in state monopolies, a public health framework for marijuana would instruct the government agency that manages the monopoly to minimize individual consumption in order to maximize public health at the population level. (Similar public health goals are explicit in Nordic alcohol monopolies [51].)

While a state monopoly is an effective approach to protect public health [51,54], in practice, however, even the strongest government monopolies for alcohol (i.e., Nordic Countries) have been eroded over time by multinational companies that argue such controls are illegal protectionism under international and regional trade agreements [4,51]. While trade agreements have been used to threaten tobacco control and other public health policies [55], clearly identifying protection of public health as the goal of the state monopoly would make it more difficult to challenge these controls, especially if sales revenues were used to help fund evidence-based demand reduction policies [49] (Table 1).

### Conclusion

It is important that jurisdictions worldwide learn from the US experience and implement, concurrently with full legalization, a public health framework for marijuana that minimizes consumption to maximize public health (Table 1). A key goal of the public health framework would be to make it harder for a new, wealthy, and powerful marijuana industry to manipulate the policy environment and thwart public health efforts to minimize use and associated health problems.
